# PDB2CD visualises dynamics within protein structures

**DOI:** 10.1007/s00249-017-1203-0

**Published:** 2017-04-03

**Authors:** Robert W. Janes

**Affiliations:** School of Biological and Chemical Sciences, Queen Mary University of London, Mile End Road, London, E1 4NS UK

**Keywords:** Protein conformation, Protein crystal structures, Protein NMR solution structures, Circular dichroism spectroscopy, Protein dynamics, Bioinformatics

## Abstract

Proteins tend to have defined conformations, a key factor in enabling their function. Atomic resolution structures of proteins are predominantly obtained by either solution nuclear magnetic resonance (NMR) or crystal structure methods. However, when considering a protein whose structure has been determined by both these approaches, on many occasions, the resultant conformations are subtly different, as illustrated by the examples in this study. The solution NMR approach invariably results in a cluster of structures whose conformations satisfy the distance boundaries imposed by the data collected; it might be argued that this is evidence of the dynamics of proteins when in solution. In crystal structures, the proteins are often in an energy minimum state which can result in an increase in the extent of regular secondary structure present relative to the solution state depicted by NMR, because the more dynamic ends of alpha helices and beta strands can become ordered at the lower temperatures. This study examines a novel way to display the differences in conformations within an NMR ensemble and between these and a crystal structure of a protein. Circular dichroism (CD) spectroscopy can be used to characterise protein structures in solution. Using the new bioinformatics tool, PDB2CD, which generates CD spectra from atomic resolution protein structures, the differences between, and possible dynamic range of, conformations adopted by a protein can be visualised.

## Introduction

A central dogma of biochemistry is that the structure of a protein is integral to its function. In order to investigate their function, protein structures have, therefore, been determined predominantly by two methods: using crystallography for the solid state, or, where possible, nuclear magnetic resonance (NMR) for the solution state. In either case, the aims have been to establish the conformation of the protein to study, for example, how a mutation might influence its function, structure, or both, or whether ligand binding modifies the structure. Many proteins have an inherent flexibility which may be a necessary feature for their function; they need to alter shape, or accommodate minor structural changes to enable ligands or other proteins to bind. On binding, the change in shape may trigger further metabolic processes for example; hence, there often has to be a balance between structural rigidity, perhaps associated with the secondary structure framework of the protein, and structural flexibility, the connecting loops, in order for proteins to perform their biological processes correctly. However, this leads to the question can such differences be identified and visualised in some simplistic way? Intrinsic to NMR studies, because the technique commonly provides a range and not a single value for the distances between atoms, an ensemble of conformations are usually generated that might be present in solution. This in itself potentially displays something of the dynamics of the conformations in solution. However, using NMR [nuclear Overhauser effect (NOE)-derived] distances data to define an ensemble of structures can generate distances weighted to shorter values than they should be which may not properly reflect the dynamics and also the true ensemble. This is usually overcome when data are both plentiful and being obtained from a number of different directions which reduces possible bias.

It might be useful to know whether one structure is the more representative of the actual solution structure within an ensemble, or perhaps the crystal structure might be more similar to the solution structure? From the ensemble of NMR structures in atomic coordinate files deposited in the Protein Data Bank (PDB; Berman et al. 2000, 2003), one structure is often denoted as either the most representative structure (usually the lowest energy structure) of the group, or the average structure from that group; in this latter case, this distinguishes it from the remaining members of the ensemble as it is not strictly a single structure generated from the data per seconds, but one generated from the structures produced, as a secondary processing step. However, neither of these structures might be the actual structure found in solution.

Circular dichroism (CD) spectroscopy is a biophysical technique used extensively for studying proteins in solution. An advantage of the technique is that it is a relatively rapid means of obtaining secondary structural information and, with well-devised experiments, it can be the key method used for monitoring changes in structure brought about by ligand binding, or by modifications to the surrounding pH or temperature. The spectrum generated from a protein will represent that of the time-averaged solution conformation of that protein if it is in a dynamic state of equilibrium in an ensemble of conformations. A comparison between the CD spectra of proteins is a simple and quick way of evaluating their conformations for similarities and differences. However, not all proteins have had their CD spectra recorded, and since there are a smaller number of CD spectra available in the Protein Circular Dichroism Data Bank (PCDDB; Whitmore et al. 2017) spectral depository relative to the numbers of protein structures available in the PDB, the PDB2CD (Mavridis and Janes 2017) package was developed whereby potential protein CD spectra can be generated from protein atomic coordinates. The package uses in combination information obtained from three structurally related levels of data to provide the generated CD spectrum; the secondary structure content [alpha helix, beta strand and “other” using the definitions from DSSP (Kabsch and Sander 1983)], the juxtapositions of these secondary structure components (their topology), and the degree of similarity of the overall folds between the query and each data set protein used. CD spectra were generated using PDB2CD from protein structures in the PDB with the aim of visualising differences that can provide useful information about the possible dynamics and overall conformations that exist in solution. These spectra are generated over the wavelength range measurable using synchrotron radiation circular dichroism (SRCD) spectroscopy (Sutherland et al. 1980; Wallace 2000; Wallace and Janes 2001), which extends to lower wavelengths than that obtained on conventional lab-based instruments, because PDB2CD was developed using spectra obtained by that technique (Lees et al. 2006).

In this study, spectra have been generated from crystal and NMR ensemble structures of the same protein using as examples predominantly alpha helical structures, alpha–beta mixed structures, predominantly beta strand structures, and proteins with low amounts of regular secondary structure content, where “other” conformations (the major component being neither alpha helix nor beta strand) are the prevalent component. This categorisation of the proteins into these four classes of structures is similar to that used in the CATH (Class - Architecture - Topology - Homology) (Sillitoe et al. 2014) classification of proteins. For each of these four classes of structural conformation, proteins were chosen with different proportions of the main secondary structure features being studied with the specific aim of showing the similarities and differences between generated spectra from such extremes. In generating these CD spectra, this not only provides a visual means of displaying conformational flexibility, but also indicates it may be feasible to use CD as a technique for identifying the conformation most likely to be present in solution.

## Methods

A select group of proteins were chosen for this study which met the following criteria. All protein crystal and solution structures compared had to arise from the same polypeptide sequence examined by each method. All structure pairs had to have either no ligands or the same ligands present. All structures had to be single-chain polypeptides. For NMR solution structures, there had to be at least 15 members within the ensemble of structures to ensure a variety of possible conformations had been explored and identified. If one member was identified as the most representative or the average structure of the ensemble, then it was indicated in the results. The chosen proteins are identified in Table 1.

**Table 1:** Chosen proteins in their four classifications for this study, their classification, name, abbreviation, crystal structure PDB code, NMR structure PDB code, number of residues, and associated references

Class of protein	Protein name	Protein abbreviation	Crystal structure PDB code	NMR structure PDB code	No. of residues	References (crystal then NMR)
Predominantly alpha helical	Globin, monomeric component M-IV	GMC	1JF4	1VRE	147	Park et al. (2002); Volkman et al. (1998)
Predominantly alpha helical	Acyl-CoA-binding protein	ACB	1HB6	2ABD	86	Van Aalten et al. (2001); Andersen and Poulsen (1993)
Mixed alpha–beta proteins	Sporulation initiation phosphotransferase F	SPI	1NAT	1FSP and 2FSP^a^	124	Madhusudan et al. (1997); Feher et al. (1997)
Mixed alpha–beta proteins	Angiogenin	ANG	1ANG	1AWZ	123	Acharya et al. (1994); Lequin et al. (1997)
Predominantly beta strand	Neocarzinostatin	NEO	1NOA	2G0K	113	Caddick et al. (2006); Teplyakov et al. (1993)
Predominantly beta strand	Ig kappa chain V-II region 26-10	IGK	1DLF	1MAK	113	Nakasako et al. (1999); Constantine et al. (1994)
Limited secondary structure	Peptidyl-prolyl Cis–Trans isomerase A	PEP	2CPL	2MZU	165	Ke (1992); Chi et al. (2015)
Limited secondary structure	Type-3 ice structuring protein HPLC 12	TIS	1GZI	1KDE and 1KDF^b^	66	Antson; Sönnichsen et al. (1996)

^a^The minimized mean structure

^b^The average structure

For this study, for each protein, the crystal structure PDB code was entered into the PDB2CD server (http://pdb2cd.cryst.bbk.ac.uk) and each generated CD spectrum was obtained. For the NMR structures, each separate model was entered into the server.

## Results and discussion

For each example protein, the results are represented in the relevant figure by a pair of panels. The first panel of the pair (part a or c in the figure) is the generated spectrum for the crystal structure in black, with the spectra for the NMR ensemble of structures in grey. Where one member of the ensemble has been identified either as the lowest energy structure or the averaged structure of the ensemble it is represented by a dashed black line. In the second panel of the pair (the “extremes panel”, part b or d in the figure), spectra are from the crystal structure (black solid line), all ensemble members with the highest overall secondary structure content (one or more dashed lines), and all ensemble members with the lowest overall secondary content (one or more dotted lines). Each example was selected to show possible differences that might be apparent in CD spectra with differing amounts of the featured secondary structure component (or components) present.

### Predominantly alpha helical proteins

**Fig. 1:**
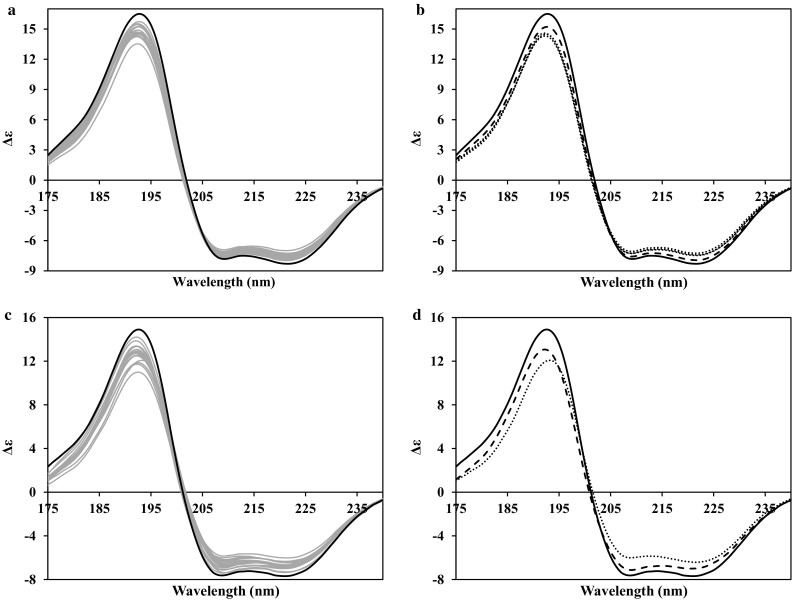
PDB2CD-generated CD spectra for predominantly alpha helical proteins; **a**, **b** globin, monomeric component M-IV (PDB codes: 1JF4, 1VRE). **c**, **d** acyl-CoA-binding protein (PDB codes: 1HB6, 2ABD). **a**, **c** NMR ensemble spectra in *grey*, crystal spectrum in *black solid*. **b**, **d** Crystal spectrum in *solid line*, largest amount of secondary structure present in the NMR ensemble conformations spectra in *dashed lines*, smallest amount of secondary structure present in the NMR ensemble conformations spectra in *dotted lines*

Figure 1 presents the generated CD spectra for two proteins where an alpha helix is the dominant secondary structure component; GMC (Fig. 1a, b) and ACB (Fig. 1c, d). In both cases, the spectra produced show the classical characteristic shape that arises where an alpha helix is the dominant component, notably negative peaks at ~222 and ~208 nm and a positive peak in the ~190 nm wavelength region. The generated spectra for both these proteins (Fig. 1a, c) indicates there is significant variation in the relative ratios of positive to negative peaks, with the wavelength positions of these peaks, and also in the ratio of their ~208 to ~222 nm negative peaks in both cases, although the overall shape of the spectrum is always retained. These variations arise most likely from the slight differences in the topology features of the structure in the ensemble, as well as the extent of the matches between the overall fold of the ensemble member and the reference structures in the database used for the PDB2CD calculation (Mavridis and Janes 2017; Lees et al. 2006). Of interest, considering the NMR ensemble spectra for both proteins in the extreme panels (Fig. 1b, d; the highest and lowest alpha helical secondary structure content for each of the ensembles), they do not represent the actual extremes of generated CD spectra in either case. So, the differences in spectral shape arise not only from differences in the secondary structure content but also from their overall conformation. For both proteins, however, when looking at the generated spectrum produced from the crystal structure, the significantly greater quantity of alpha helix calculated as present from DSSP (Kabsch and Sander 1983) is clearly the reason behind these spectra having a greater magnitude than those of their NMR ensemble structures.

### Mixed alpha–beta proteins

**Fig. 2:**
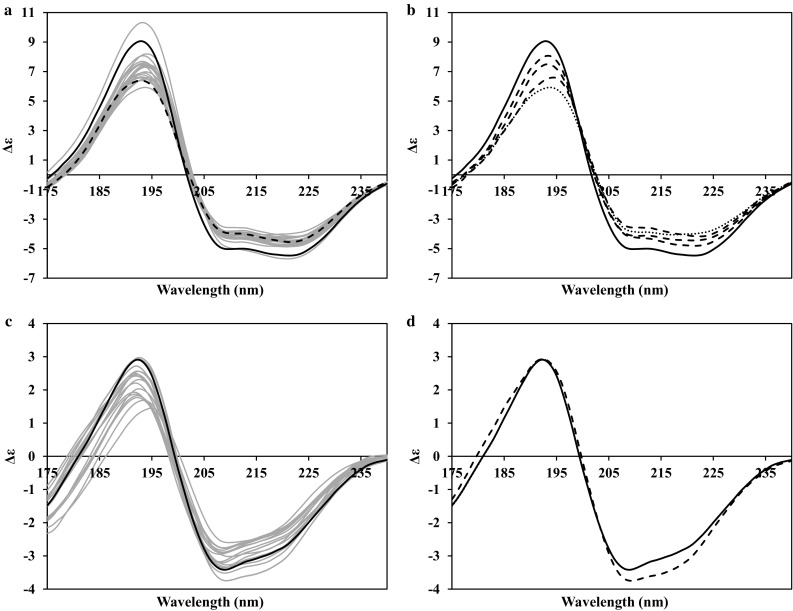
PDB2CD-generated CD spectra for mixed alpha–beta proteins; **a**, **b** sporulation initiation phosphotransferase F (PDB codes 1NAT, 1FSP and 2FSP. **c**, **d** Angiogenin (PDB codes: 1ANG, 1AWZ). **a**, **c** NMR ensemble spectra in *grey*, crystal spectrum in *black solid*. **a** Minimized mean NMR structure in *black dashed*. **b**, **d** Crystal spectrum in *black*, largest amount of secondary structure present in the NMR ensemble conformations spectra in *dashed*, smallest amount of secondary structure present in the NMR ensemble conformations spectra in *dotted lines* (almost coincident with the *crystal line* in **d**)

Figure 2 presents the CD spectra generated by PDB2CD for two mixed alpha–beta proteins, SPI and ANG. These two examples are from opposite poles of the alpha–beta region of structures: SPI has considerably more alpha helix content than ANG in their crystal structures (alpha 47.9% beta 22.7% against alpha 20.3% beta 31.7%, respectively). This is also seen in their NMR ensemble structures as well in the shapes of the spectra generated for each protein. In Fig. 2a, b, the spectra for SPI, all have recognisable alpha helical characteristics; however, they have noticeably smaller magnitudes and a reduced ratio between the negative and positive peaks. There is also a greater diversity in the wavelength maximum positions for the ~190 nm peak; also, the negative peaks at ~222 nm are larger than the ~208 nm peaks in almost all cases. In Fig. 2a, the spectrum for the minimized average structure of the ensemble is shown as a dashed line. This spectrum is clearly not the average spectrum of those generated for the ensemble, demonstrating that the methods used in PDB2CD are each being weighted according to the degree of match to their specific features and, therefore, the resultant spectrum generated arises from the overall structural properties as a result.

In contrast to Fig. 2a, the spectra in Fig. 2c for ANG have substantially reduced magnitudes for their peaks and in the ~222 nm wavelength region, there is only a negative shoulder present in all spectra; both observations are consistent with increased beta content and reduced alpha content. Additionally, the positive and negative peaks are nearly equal in magnitude. For both alpha–beta-mixed proteins, the crystal structure spectrum is now not the overall maximum spectrum generated within each group; at least one spectrum from the NMR ensemble has one or more peaks of larger magnitude. For the alpha helical proteins previously discussed, the maxima peaks are representative more of secondary structure content present in the conformation than their overall topology. However, the maxima in these mixed alpha–beta proteins arise not only from the secondary structure content, but the juxtapositions of these features also contribute to the spectra produced, carrying more relative weight in generating the CD spectra. This is most evident in Fig. 2d for ANG where the generated spectrum for the crystal structure almost matches that for the structure with the least overall secondary structure content (their total secondary structure content differs by more than 3% in beta sheet but their alpha helix contents are identical). These spectra arise from a combination of all three methods used in PDB2CD where the overall results probably use the same database proteins to generate the spectra with only slightly different weightings in each case leading to comparable resulting spectra.

### Predominantly beta strand proteins

**Fig. 3:**
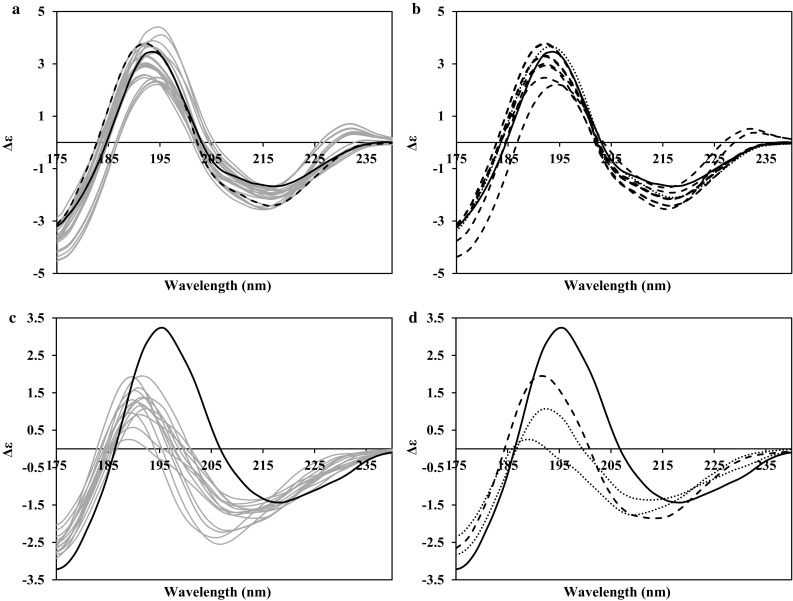
PDB2CD-generated CD spectra for predominantly beta strand proteins; **a**, **b** neocarzinostatin (PDB codes: 1NOA, 2G0K). **c**, **d** Ig kappa chain V-II region 26-10 (PDB codes: 1DLF, 1MAK). **a**, **c** NMR ensemble spectra in *grey*, crystal spectrum in *black solid*. **a** Minimized mean NMR structure in *black dashed*. **b**, **d** Crystal spectrum in *black*, largest amount of secondary structure present in the NMR ensemble conformations spectra in *dashed*, smallest amount of secondary structure present in the NMR ensemble conformations spectra in *dotted lines*

Figure 3 shows the CD spectra generated for two predominantly beta strand proteins; NEO and IGK. In these cases, the dominant secondary structure feature is beta strand in that there is little to no alpha helical content in either structure. Spectra arising from beta strands are significantly smaller in magnitude than those from alpha helical proteins with comparable percentages of secondary structure and this is seen in Fig. 3. In addition, there is a greater diversity of spectral characteristics displayed by beta strand structures due, for example, to their topological arrangement as either parallel, or anti-parallel sheets, or a mixture of both. Often there is a further added feature in that there is an in-built twist within the sheet away from planarity (Ho and Curmi 2002; Wallace 2009; Micsonai et al. 2015). This pair of proteins have CATH (Sillitoe et al. 2014) classifications of 2.60.40.230 and 2.60.40.10, matching the class (mostly beta strand proteins), architecture (the strands into sheet forms), and the topology (the juxtapositions of those sheets) level. The aim of these examples was to illustrate the similarities and differences between the generated CD spectra when there is an overall structural similarity but a difference in the quantity of beta strand present in the structures. For NEO, most of the NMR ensemble structures (42 of 47) contain beta strand contents of over 50 percent, while “other” [the sum of the residues not classified as alpha helix or beta strand using the DSSP (Kabsch and Sander 1983) criteria] is the main component for the IGK. Therefore, in these two examples, it might be expected that there should be some degree of similarity between the sets of CD spectra produced reflecting the nature of the secondary structure present and the character of the topology of that structure, but that differences would result given the different proportions of beta strand present.

The generated spectra for NEO in Fig. 3a, b appear to group into two broad classes: the first is reasonably exemplified by the most representative member of the ensemble in the dashed spectrum in Fig. 3a, while the second group has a unique slightly positive peak at ~230 nm, a negative peak at ~215 nm and a positive peak at ~192 nm, each being of lesser magnitude than the other group of spectra. Interestingly, a number of members in the ensemble possess the largest beta strand content (54%), indicated by the dashed lines in Fig. 3b, yet these fall into both groups of spectra which indicates these groups are being generated primarily by topological and full structural comparison differences within the ensemble rather than only secondary structure content. Once again this is similar to the examples for the mixed alpha–beta proteins shown in Fig. 2. The crystal structure of NEO has less regular secondary structure content (49.6% beta strand) than does the most representative of the ensemble NMR structures (54%) and a slight difference in the topology of these beta strands; also, it is these features that result in the distinctions between the two generated CD spectra.

The IGK has substantially less beta strand content for the NMR structures than does NEO (the average for the ensemble is 36.9%, compared with 52.2%). Again, this reduces the magnitudes of the peaks generated, although their overall shape does resemble those of the other protein because they are identical in CATH classification to the topology levels. The ensemble of NMR structures generates a wide range of CD spectra (relative to the overall magnitude, Fig. 3c) which is reflective of the fact that there is less beta strand content in the structures (the ensemble range is 33.6–40.7%), resulting in a greater influence from both the relative topology of this structure and also the remaining non-canonical content in the conformations present. In Fig. 3d, the two spectra generated from the NMR structures with the least amount of beta strand secondary structure content (the dotted lines) differ in their appearance because of differences in their topology and overall structure.

### Limited secondary structure proteins

**Fig. 4:**
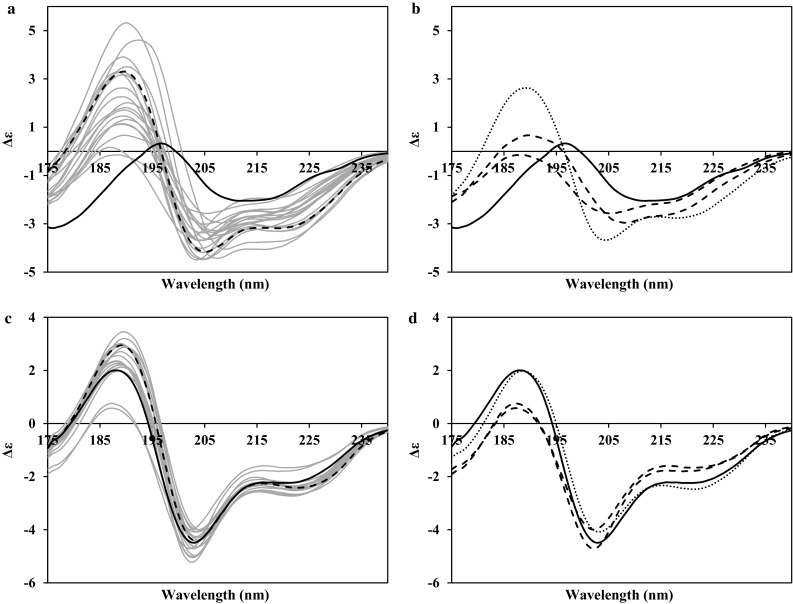
PDB2CD-generated CD spectra for limited secondary structure proteins; **a**, **b** peptidyl-prolyl cis–trans isomerase A (PDB codes: 2CPL, 2MZU). **c**, **d** Type-3 ice-structuring protein HPLC 12 (PDB codes: 1GZI, 1KDE and 1KDF). **a**, **c** NMR ensemble spectra in *grey*, crystal spectrum in *black solid*. **a** Minimized mean NMR structure in *black dashed*. **c** Averaged NMR structure in *black dashed*. **b**, **d** Crystal spectrum in *black*, largest amount of secondary structure present in the NMR ensemble conformations spectra in *dashed*, smallest amount of secondary structure present in the NMR ensemble conformations spectra in *dotted lines*

Only a few protein structures in the PDB are comprised of small amounts of secondary structure components probably because they are not regularly ones to crystallise. There are even fewer structures where the conformation has been determined by both NMR solution and X-ray structure methods. Two examples have been chosen from this small group which represent again two potential extremes of the genre; their calculated spectra are given in Fig. 4. The first, PEP, has limited amounts of both alpha helix and beta strand, which, in the NMR ensemble, usually sum to less than 30% of the secondary structure content of the protein (in 15 of the 20 structures). Note that in contrast to this, the crystal structure of the same protein has more than 41% secondary structure in its conformation. The second of the two examples, TIS, also has limited amounts of regular secondary structure components represented in the ensemble; none of them, including the average structure calculated from the data, has any alpha helical content, and most have beta strand contents under 10% (18 out of 23 structures). The comparable crystal structure has ~6% alpha helix and ~15% beta strand.

In Fig. 4a, it is clear that in this type of protein, the CD spectra generated are highly susceptible to small differences in their total component content, the topology of these components and their overall matching to other structures within the PDB2CD data set. The best representative structure in the ensemble, denoted by the first entry of the PDB file and indicated by a dashed spectrum in Fig. 4a, has a generated CD spectrum with a negative shoulder at ~222 nm, a negative peak at ~205 nm and a positive peak at ~190 nm, where the negative peak has a greater magnitude than does the positive peak. A number of the ensemble members also have these overall characteristics, but there is considerable variation within the group of spectra as to the preservation of the shoulder, their magnitudes, the relative ratios between their magnitudes of the peaks, and wavelength positions of these peaks. Focussing on the “extreme” spectra in Fig. 4b shows that the structure in the ensemble with the lowest quantity of secondary structure content, the dotted line spectrum, has a spectral appearance similar to that of the representative structure of the ensemble in Fig. 4a only with lower overall magnitudes. The spectrum generated for the crystal structure, with a substantially higher relative secondary structure content, is significantly different, having essentially a broad negative plateau between ~210 and ~218 nm, a positive peak at ~197 nm, that is substantially lower in magnitude than the higher wavelength negative peaks, and there is a larger magnitude negative peak at ~176 nm. It is clear that different protein structures, and, hence, CD spectra, are contributing to this generated spectrum in contrast to those used in the majority of the spectra for the ensemble of structures. The two ensemble structures with the highest secondary structure content, represented by the dashed lines in Fig. 4b, appear to have spectral properties that are combinations of features from those of the lowest regular secondary structure content spectrum and the crystal spectrum, falling in both cases, somewhere between these spectra in shape.

When considering the generated spectra for the second example with minimal secondary structure components present (Fig. 4c), almost all have similar shapes: a shallow negative peak or shoulder at ~223 nm, a more pronounced negative peak at ~205 nm, and a positive peak at ~189 nm, which has lower magnitude than the negative peak. This is exemplified by the CD spectrum generated for the average structure derived from the ensemble (dashed line Fig. 4c), while the crystal structure spectrum has more of a shoulder at ~223 nm than a peak. There are two spectra from the ensemble of structures that are very different from the other spectra, and in Fig. 4d, it is clear these both arise from the highest secondary structure content members. Both spectra are smaller in magnitude at the negative ~223 nm region, one having a shoulder, whilst the other has a very shallow peak. At the negative ~205 nm wavelength, there is notable difference between them; one has a sharper peak with magnitude slightly larger than that of the crystal structure spectrum; the other has a broader shallower peak more comparable in magnitude to that of the spectrum from the member with the lowest secondary structure content. In the positive peak region, ~189 nm, both have substantially smaller magnitudes than any of the other generated spectra. Of note, the spectrum generated for the member with the lowest secondary structure content (dotted line Fig. 4d), is derived solely from matches between the tertiary structures of the query spectrum and the SP175 data set used in PDB2CD as there is no “secondary structure content” (here, meaning no alpha helix or beta strand); hence, no contribution is being made from either this factor or the topology between such content. In addition, for the structures with minimal secondary structure content, the generated spectra in Fig. 4b, d have larger positive and negative peaks than spectra from some structures with more secondary structure content. This suggests that for structures with limited secondary structure content, the CD spectra generated are more strongly influenced by the topology and/or matches between tertiary structures features than by the secondary structure content.

## Conclusions

Protein structures obtained from either NMR or crystallographic studies can be used by PDB2CD to generate CD spectra as a novel bioinformatics approach for visually revealing the different natures of these differing structures. When alpha helix content dominates the protein structure, all the spectra generated from the two methods appear to differ mostly in magnitude, but not significantly in shape. Mixed alpha–beta structures generate spectra dependent upon the quantity of helix present; a larger helical content produces characteristics of an alpha helical spectrum more than when a smaller proportion of helix to sheet content is present. The CD spectra with the largest diversity generated come from structures dominated by beta content or from structures with very little canonical secondary structure content present. Now, it is the topology of these features that plays a more significant role as does the extent of structural overlap between the query protein and those in the PDB2CD data set used. Notably, however, for proteins with a relatively high degree of conformational similarity within an ensemble of NMR structures, or an additional crystal structure, the CD spectra generated retain some comparable features, but do show discrete differences one from another. These differences could also be valuable in cases where a sparsity of NOE data might produce a bias in the distance data used to generate the ensemble of structures, resulting in some regions have shorter distances than they should have. In these cases, the CD spectra generated could provide evidence for such structures being problematic.

This study has demonstrated that PDB2CD-generated CD spectra could be employed to identify whether a protein exists in solution as a single structure or as a dynamic state in equilibrium between a few of the possible ranges of conformations such as those identified by NMR. Collecting an experimental CD spectrum would enable direct comparison between it and the generated spectra. Finding the closest match between either a single generated spectrum or a combinatorial summation of generated spectra and the experimental CD spectrum would determine the nature of the structure in solution.
